# Expertise Shapes Multimodal Imagery for Wine

**DOI:** 10.1111/cogs.12842

**Published:** 2020-05-07

**Authors:** Ilja Croijmans, Laura J. Speed, Artin Arshamian, Asifa Majid

**Affiliations:** ^1^ Faculty of Social and Behavioral Sciences Utrecht University; ^2^ Centre for Language Studies Radboud University; ^3^ Department of Clinical Neuroscience Karolinska Institutet; ^4^ Department of Psychology Stockholm University; ^5^ Department of Psychology University of York

**Keywords:** Imagery, Olfaction, Vision, Taste, Wine expertise, Training

## Abstract

Although taste and smell seem hard to imagine, some people nevertheless report vivid imagery in these sensory modalities. We investigate whether experts are better able to imagine smells and tastes because they have learned the ability, or whether they are better imaginers in the first place, and so become experts. To test this, we first compared a group of wine experts to yoked novices using a battery of questionnaires. We show for the first time that experts report greater vividness of wine imagery, with no difference in vividness across sensory modalities. In contrast, novices had more vivid color imagery than taste or odor imagery for wines. Experts and novices did not differ on other vividness of imagery measures, suggesting a domain‐specific effect of expertise. Critically, in a second study, we followed a group of students commencing a wine course and a group of matched control participants. Students and controls did not differ before the course, but after the wine course students reported more vivid wine imagery. We provide evidence that expertise improves imagery, exemplifying the extent of plasticity of cognition underlying the chemical senses.

## 1 Introduction

There are considerable individual differences in the ability to imagine. Experts—individuals with in‐depth knowledge and abundant practical experience in a domain (Ericsson, Prietula, & Cokely, 2007; Weinstein, 1993)—have more vivid imagery. Expert abacus users, for whom visual imagery is pertinent, are better at imagining pictures than novices (Hatta & Miyazaki, 1989); while perfumers report more ease in evoking odor images (Gilbert, Crouch, & Kemp, 1998; Plailly, Delon‐Martin, & Royet, 2012), reflected also in functional changes in brain regions involved in odor imagery (Plailly et al., 2012). Similarly, in a task where participants have to choose which of two stimuli best matches a third, chefs respond faster when imagining similarities between fruit smells, while musicians are faster imagining similarities between musical instrument timbres (Bensafi et al., 2017; Bensafi, Tillmann, Poncelet, Przybylski, & Rouby, 2013). Together these studies suggest experts are better than novices at imagery across sensory modalities, including smell.

The fact that experts are better at olfactory imagery is interesting because previously it has been claimed that the average person simply cannot imagine odors at all (Arshamian & Larsson, 2014; Stevenson & Case, 2005). In fact, there appears to be a general asymmetry in the ability to conjure perceptual imagery: The self‐reported frequency of mental imagery differs across modalities, with vision being imagined most often, and smell and taste least often (Lawless, 1997). Similarly, there are differences in the vividness of self‐generated imagery. When people are able to conjure an image, they report it is most vivid for vision, followed by sound and touch, with smell and taste imagery being the least vivid (Andrade, May, Deeprose, Baugh, & Ganis, 2014). This makes cases of smell and taste imagery in experts particularly interesting to probe in detail, since it suggests this asymmetry between the senses may not be cast in stone (cf. Majid et al., 2018).

Differences in imagery between experts and novices are usually interpreted as the product of training and practice. For example, motor imagery training has been found to improve the ability to visualize skating movements in ice skaters (Rodgers, Hall, & Buckolz, 1991). Similarly, music training improves imagery for the pitch of song notes and acoustic character of everyday sounds, but not visual imagery for objects (Aleman, Nieuwenstein, Böcker, & de Haan, 2000). Such studies suggest imagery ability is honed by learning.

However, current studies cannot rule out pre‐existing differences between good and poor imaginers. A genetic predisposition may prompt someone to select a profession in which their abilities are rewarded, for example. In other words, the ability to engage in olfactory and gustatory imagery may predispose someone to become a wine expert. It is clear that there are common neurobiological traits between some experts. Specific genetic variants are predictive of musical aptitude and creativity (e.g., Oikkonen et al., 2014), and music expertise is strongly heritable (Mosing, Madison, Pedersen, Kuja‐Halkola, & Ullén, 2014). Similarly, the ability to taste the bitter substance PROP—a genetically predetermined phenotype—is found more often in wine experts than the general population (e.g., Hayes & Pickering, 2011).

So either experts are better imaginers in the first place, and so become experts; or through training and practice, experts learn skills that enable them to become better at imagining. Existing cross‐sectional studies comparing imagery abilities cannot tease apart the role of learning versus pre‐existing dispositional differences. Here we test these two possibilities with wine experts in the multisensory domain of wine. We ask for the first time whether wine experts have superior imagery for wine; and, if so, whether this ability is learned.

Before testing the role of learning, it is important to first establish whether wine experts do, indeed, have better imagery for wines. Some researchers have been skeptical about whether wine experts really have exceptional abilities (e.g., Quandt, 2007). So we cannot simply assume sensory imagery for wines is better in wine experts without specifically testing for it. At the same time, there are good reasons to think imagery would be better in this cohort. Wine experts have to be able to represent the colors and odors—as well as flavor—of wine with acuity (Shepherd, 2015; Tempere, de Revel, & Sicard, 2019). Typically an expert will begin by visually inspecting a wine for clarity and color. Next, the wine will be swirled and sniffed; and only then will it be tasted. In addition, wine experts compare the wine at hand with memorized prototypical wines (Parr, 2018), so as to appreciate and describe a fine wine. Language can direct an expert's attention to specific components of a wine (cf. Goldstone, 1998; Majid, Bowerman, Kita, Haun, & Levinson, 2004), making certain odors and flavors more salient. Honing attention in this way could further affect how wine is remembered and imagined (cf. White, Thomas‐Danguin, Olofsson, Zucco, & Prescott, 2020). So, a priori, imagery ought to play a pertinent role in wine expertise (Tempere et al., 2019), although the critical empirical evidence is lacking.

We began by first testing whether wine experts have more vivid imagery for the color, odor, and taste^1^ of wines than novices. To this end, we used the recently constructed and validated Vividness of Wine Imagery Questionnaire (VWIQ; Croijmans, Speed, Arshamian, & Majid, 2019). This questionnaire presents individuals with different scenes (e.g., a wine tasting, a dinner in a restaurant) that participants were instructed to imagine, and then to rate for vividness of the corresponding imagery of the featured wine.

Critically, in a second study, we implemented a longitudinal design where we followed students enrolled in a training course to become vinologists. Students and matched controls completed the VWIQ at two time points, for students before and after training. We were thus able to determine whether differences in imagery ability are precursors to becoming a wine expert or whether imagery is learned through training.

## 2 Study 1: Are wine experts better at imagining the multisensory aspects of wine?

### 2.1 Participants

A total of 146 Dutch participants, all living in the Netherlands at the time of the experiment, were recruited for this study. All participants self‐reported normal olfactory functioning, and in case of a cold or hay‐fever, testing was postponed until the participant was well. Previous studies testing olfactory imagery in experts have employed relatively smaller sample sizes (e.g., Bensafi et al., 2017, *n* = 13 professional cooks; Plailly et al., 2012, *n* = 14 student perfumers, *n* = 14 professional perfumers). Our expert sample size, which was based on the availability of experts, was five times larger. There were 66 wine experts (20 female; *M*_age_ *= *48.7 years old, range 21–70). They were either experienced professionals in the field of wine (e.g., vinologists, certificated sommeliers) or amateur connoisseurs with an attested interest in wine (e.g., had an extensive wine collection or a vineyard) following previous criteria of what constitutes a wine expert (Melcher & Schooler, 1996). These experts were recruited by actively approaching experts in stores and through e‐mail and phone, as well as via word‐of‐mouth. In addition, a few experts responded to a call placed in a magazine.

Sixty‐six Dutch novices were matched to the experts in age and gender (20 female; *M*_age_ = 49.0 years old, range 24–70). The remaining 14 participants were excluded from analyses since they did not meet the expert criteria outlined above, but could not be considered wine novices either (*n* = 3). Others were excluded because experts and novices were recruited and tested in parallel, and novices could not be yoked to a wine expert based on age or gender (*n* = 11). A *t* test confirmed that the expert and novice group in the final sample did not differ in age, *t*(130) = 0.21, *p* = .836.

To establish whether groups differed on wine expertise, the Wine Knowledge Test (WKT; Croijmans & Majid, 2016) was administered. This questionnaire consists of 15 questions about wine, for example “What is the traditional color of wine made of chardonnay grapes?” and “What is the difference between aroma and bouquet?” The responses showed high internal consistency, McDonald's ω = 0.854. The questionnaire confirmed wine experts (*M* = 13.61, *SD* = 1.2) had significantly higher wine knowledge than novices (*M* = 7.91, *SD* = 2.2), *t*(130) = −18.49, *p* < .001, *d* = 3.2.

### 2.2 Materials

#### 2.2.1 Wine imagery

We administered the Dutch version of the VWIQ (see Croijmans et al., 2019). The VWIQ is composed of six scenarios related to wine assessing the vividness of the imagined wine for color, smell, and taste; for example, “Imagine you are going to a short wine tasting where you will try different wines. The tasting starts with a French white wine, a Sauvignon Blanc,” with each scenario followed by three 5‐point rating scales (ranging from “1—no image at all, just knowing that I am thinking about the object” to “5—perfectly clear and as vivid as the real situation”). The VWIQ ratings were averaged across scenarios by perceptual modality: color (VWIQ‐C), odor (VWIQ‐O), and taste (VWIQ‐T). Scores on each subscale ranged from 1 (low imagery vividness) to 5 (high imagery vividness). The questionnaire showed high internal consistency, McDonald's ω = 0.916.

#### 2.2.2 Imagery for everyday odors

Since there is controversy over the existence of odor imagery in the field, we also administered a Dutch version of the established odor imagery questionnaire (i.e., the VOIQ; Gilbert et al., 1998). This questionnaire furthermore allows for a test of the domain specificity of wine expertise: Since wine experts are preoccupied with their sense of smell, they may also improve their general odor imagery ability. In contrast hand, if wine expertise is truly domain specific (i.e., restricted to the domain of wine), wine experts should not report more vivid imagery for common everyday odors, but only for wine. The VOIQ contains 16 statements describing olfactory scenes (e.g., “The smell of your shirt or blouse when you remove it”). Participants were instructed to imagine each scene and rate how vivid their mental image was using the same 5‐point scale as the VWIQ. However, the VOIQ scale scores are reversed compared to the VWIQ (with scores ranging from “1—perfectly clear and as vivid as the real situation” to “5—no image at all, just knowing that I am thinking about the object”), so all VOIQ question scores were reverse scored; that is, higher scores indicated more vivid imagery in both questionnaires. The VOIQ is scored by averaging across the 16 questions with final scores ranging between 1 (low imagery vividness) and 5 (high imagery vividness). The internal consistency of this questionnaire was high, McDonald's *ω* = 0.907.

### 2.3 Procedure

Written consent was obtained before the experiment began. Participants completed the three questionnaires during a break of a different experiment also involving wine. The questionnaires were completed using paper and pencil, and they were always completed in the same order: VWIQ, VOIQ, WKT. Both Study 1 and Study 2 were approved by the Ethics Assessment Committee, Humanities Lab at Radboud University, and carried out in accordance with the provisions of the World Medical Association Declaration of Helsinki.

### 2.4 Results

To test the hypothesis that experts are better than novices at imagining wine, the scores on the individual modalities of the VWIQ were compared between groups using a mixed ANOVA, with Modality (three levels: color, odor, and taste) as a within‐participants factor and Expertise (two levels: wine experts and novices) as a between‐participants factor. Corrections in the degrees of freedom for sphericity assumption violations were applied where appropriate.

**Fig. 1:**
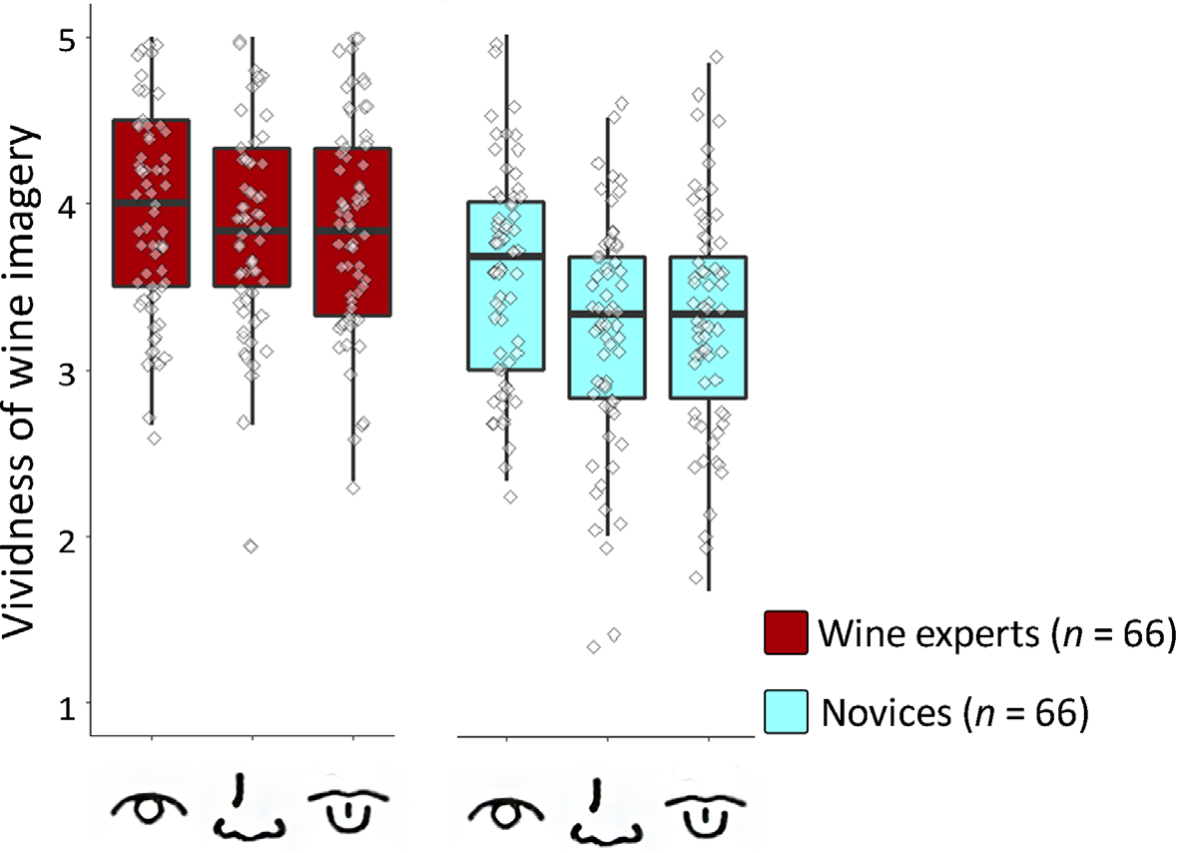
Boxplots displaying mean VWIQ scores per modality for wine experts and novices for Study 1. Note: Box‐and‐whisker plots display median, first and third quartiles ranges, and whiskers indicate range of the data. Diamonds portray individual data points. Icons represent the imagery modalities: color, smell, and taste.

Wine experts reported more vivid imagery for wines overall, *F*(1, 130) = 28.93, *p* < .001, $\eta_{p}^{2}$ = 0.18. In addition, there was a main effect of Modality, *F*(2, 260) = 15.02, *p* < .001, $\eta_{p}^{2}$ = 0.10. This main effect must be interpreted in the context of a significant interaction between Modality and Expertise, *F*(2, 260) = 5.16, *p* = .006, $\eta_{p}^{2}$ = 0.04. Pairwise comparisons showed that for novices, visual imagery (*M* = 3.58, *SD* = 0.62) was more vivid than taste imagery (*M* = 3.30, *SD* = 0.67), *p* = .002, *d* = 0.59 and odor imagery (*M* = 3.17, *SD* = 0.73), *p* < .001, *d* = 0.46; and taste imagery, in turn, was more vivid than odor imagery, *p* = .04, *d* = 0.14. In contrast, wine experts showed no difference in vividness of imagery across modalities, *p*s > .05 (see Fig. 1). In addition, wine experts had more vivid imagery than novices for each modality separately (*p*s < .01).

**Fig. 2:**
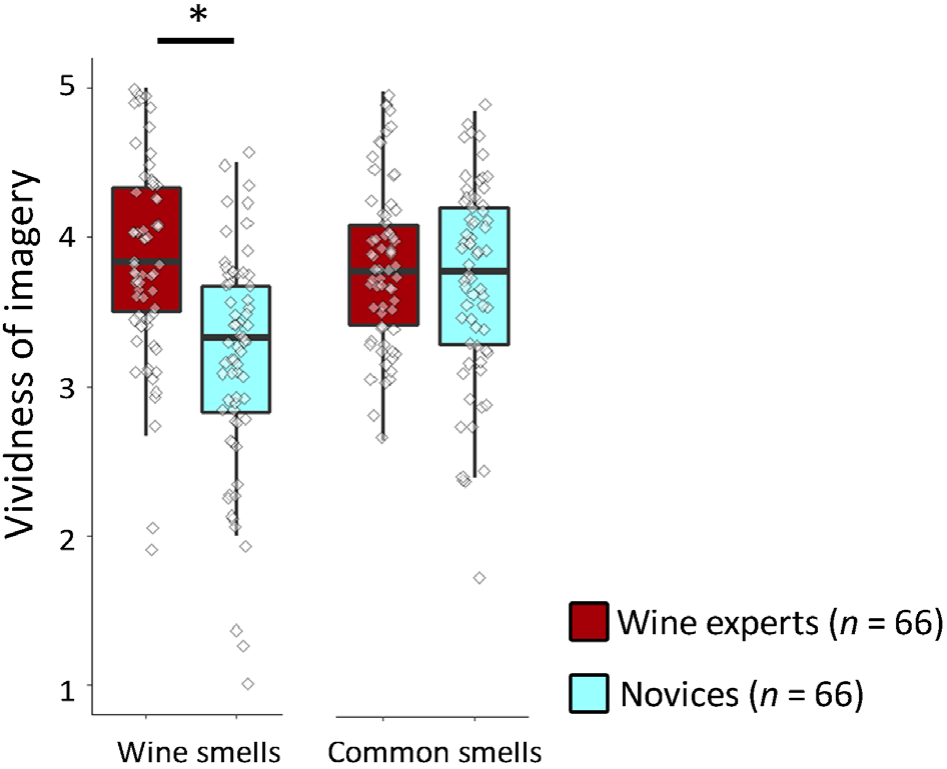
Boxplots displaying imagery vividness scores for wine smells (VWIQ) and common smells (VOIQ), for wine experts and novices, for Study 1. Note: Box‐and‐whisker plots display median, first and third quartiles ranges, and whiskers indicate range of the data. Diamonds portray individual data points. Asterisk (*) denotes a significant difference with *p* < .001.

Wine experts did not, however, report enhanced imagery for everyday odors (see Fig. 2). Using an Expertise (wine experts vs. novices) by Odor type (wine odors vs. everyday odors) ANOVA, scores on the VWIQ‐smell subscale and VOIQ were compared between the two groups. Wine experts reported overall more vivid imagery, evidenced by a main effect of Expertise, *F*(1, 130) = 16.09, *p* < .001, $\eta_{p}^{2}$ = 0.110. There was a main effect of Odor type, *F*(1, 130) = 19.64, *p* < .001, $\eta_{p}^{2}$ = 0.131, but there was also a significant interaction, *F*(1, 130) = 28.34, *p* < .001, $\eta_{p}^{2}$ = 0.179. Pairwise comparisons showed wine experts reported more vivid imagery for wine odors (*M* = 3.84, *SD* = 0.65) than novices (*M* = 3.17, *SD* = 0.73), *p* < .001, *d* = 0.97, but there was no difference in vividness for imagery of everyday odors between experts (*M* = 3.80, *SD* = 0.55) and novices (*M* = 3.68, *SD* = 0.65), *p* = .245, *d* = 0.20.

We show for the first time that wine experts have more vivid multisensory imagery for wines than novices. Novices' vividness of imagery followed the previously attested hierarchy of sensory imagery (Andrade et al., 2014; Lawless, 1997), with more vivid imagery for visual aspects of wine (i.e., the color), than for the taste or odor—modalities that are also traditionally regarded as difficult to imagine, and paralleling findings on olfactory vs. visual language, for example (e.g., Majid et al., 2018). But this asymmetry was absent in wine experts: wine experts had equally vivid imagery for all modalities. Importantly, the more vivid imagery of experts was restricted to wine; wine experts did not differ from novices in their imagery for everyday odors.

## 3 Study 2: Is expert imagery learned or innate?

To assess whether the enhanced multisensory wine imagery displayed by experts in Study 1 is learned or innate, we measured wine imagery in budding wine experts before formal training and 6 months into an intensive course on wine, and compared their behavior to a matched control sample. One major factor to take into consideration is that people seeking to become a wine expert already have an initial interest in wine. So we would expect wine students and controls might differ in basic, factual knowledge about wines. Critically, however, if wine imagery draws on elaborated perceptual and cognitive training learned through a practical wine course, then wine students should only show enhanced imagery after such training.

### 3.1 Participants

Participants studying for “registervinoloog” (*registered vinologist,* Dutch SDEN 4, comparable to WSET level 4^2^) were recruited from a cohort of new students from the Dutch “Wijnacademie” (*Wine Academy* ^3^). This training consists of several courses over 6 months, involving weekly theory sessions in the morning and practical sessions recognizing and distinguishing wines in the afternoon. Students are estimated to have tasted roughly 600–800 different wines over the course of 6 months.

To be able to detect a medium effect (*f* = 0.25) with a statistical power of .80 for a mixed interaction, the targeted sample size was 82. All students (approximately 50) who enrolled in the course were invited to take part in the study. We were able to recruit 77 participants in total. Thirty‐two students (15 women; *M*_age_ *= *43.6 years, range 22–66) agreed to participate, 14 of whom reported having a background in the food and beverage industry, for example working in a restaurant (*n = *5), working as a coffee roaster (*n* = 2), or working in the food department of a supermarket or deli (*n* = 7). All students had successfully followed entry‐level courses in wine. Twenty students (i.e., 62.5%; 9 women; *M*_age_ *= *45.5 years, range 27–63) also completed the survey at time 2 (T2). A group of 45 control participants (30 women; *M*_age_ *= *40.4 years, range 25–64) with little experience of wine other than being occasional consumers were also recruited. Of this group, 37 (82.2%) also completed the survey after 6 months, at T2 (25 women; *M*_age_ *= *41.1 years, range 25–64). In the group of participants who completed the questionnaires both at T1 and T2, there was no significant difference in age, *t*(55) = 1.18, *p* = .211, or gender, χ^2^(1) = 2.75, *p* = .097.

### 3.2 Materials

At T1 the VWIQ, VOIQ, and WKT were administered. Internal consistency of these questionnaires was high (ω_VWIQ_ = 0.946; ω_VOIQ_ = 0.917; ω_WKT_ = 0.888). In addition, participants reported their wine expertise, using the Self‐rated Wine Expertise Questionnaire (SWEQ, Dutch translation from Johnson & Bastian, 2007). This five‐item questionnaire asks participants to compare their knowledge of wine to peers in different situations (e.g., “Among my circle of friends, I am one of the ‘experts’ on wine”) on a scale ranging 1 (*strongly disagree*) to 9 (*strongly agree*). The SWEQ also had high internal consistency, McDonald's ω = 0.922. Finally, because the control participants needed to be smell and flavor novices, we asked all participants to report their background involvement in the food and beverage industry, using open questions (“Have you followed any courses related to food, drinks, or smells and tastes in general? Think for example of courses in the area of coffee, tea, chocolate, cooking courses, perfume workshops, etc. And if so, please specify these here.”). These questions were checked for aberrations (e.g., whether someone may be considered a wine expert in the control group), but otherwise they were not analyzed.

The follow‐up at T2 consisted of the same questionnaires as administered in T1, with the addition of the Vividness of Visual Imagery Questionnaire (VVIQ; Marks, 1973). This was included as an additional test of imagery across modalities. Since we expected wine training to only affect wine imagery, we hypothesized no differences for visual imagery for everyday scenes (following the results of the VOIQ in Study 1). The VVIQ was completed twice: once with eyes open and once with eyes closed. The VVIQ attested high internal consistency, ω_eyes open_ = 0.937; ω_eyes closed_ = 0.949. In addition, at T2 participants reported how much time they spent per week tasting wine; and how many different wines and glasses of wine per week they had tasted in the past year.

### 3.3 Procedure

Participants received a letter explaining the procedure before taking part in the study, and signed to confirm they understood and confirmed their participation was voluntary. All participants received a gift voucher of €10 for each test session. Students completed the first survey using paper and pencil during the first month of the 9‐month course they were enrolled in and completed the second survey online using Qualtrics after 6 months. Controls completed both surveys online using Qualtrics, with approximately 6 months between both measurements.

### 3.4 Data processing

VWIQ, VOIQ, and WKT scores were treated in the same way as in Study 1, and an average VVIQ score (averaging eyes‐closed and eyes‐open scores) was also calculated. The SWEQ questions 2, 4, and 5 were reverse scored, and all five items then summed into a subjective wine expertise score.

Only data from participants who completed the questionnaire at both times were included (*n* = 57). One participant from the student group was excluded as they were an outlier on several of the questionnaires (following the outlier criterion *M* ± 2.5 *SD*)*.*

### 3.5 Results

As expected, students and controls differed in their wine knowledge even before training according to both the WKT and SWEQ. Using ANOVA, with Group (students, controls) as a between‐participant factor, and Time (T1, T2) as within‐participant factor, we found a main effect of Group on wine knowledge (WKT): Students (*M* = 13.68, *SD* = 0.75) had significantly higher scores than controls (*M* = 7.81, *SD* = 2.53), *F*(1, 55) = 102.18, *p* < .001, $\eta_{p}^{2}$ = 0.650. There was no effect of Time, *F*(1, 55) = 1.26, *p* = .267, $\eta_{p}^{2}$ = 0.022, and no interaction between Group and Time on wine knowledge scores, *F*(1, 55) = 1.26, *p* = .267, $\eta_{p}^{2}$ = 0.022. Students also rated their own wine expertise (*M* = 36.45, *SD* = 3.40) significantly higher than controls (*M* = 16.43, *SD* = 8.97), *F*(1, 55) = 91.79, *p* < .001, $\eta_{p}^{2}$ = 0.625. There was no effect of Time, *F*(1, 55) = 0.01, *p* = .942, $\eta_{p}^{2}$ = 0.000, and no significant interaction between Group and Time, *F*(1, 55) = 3.00, *p* = .089, $\eta_{p}^{2}$ = 0.052. So, on these measures of factual wine knowledge and self‐reported knowledge, students were at ceiling.

**Fig. 3:**
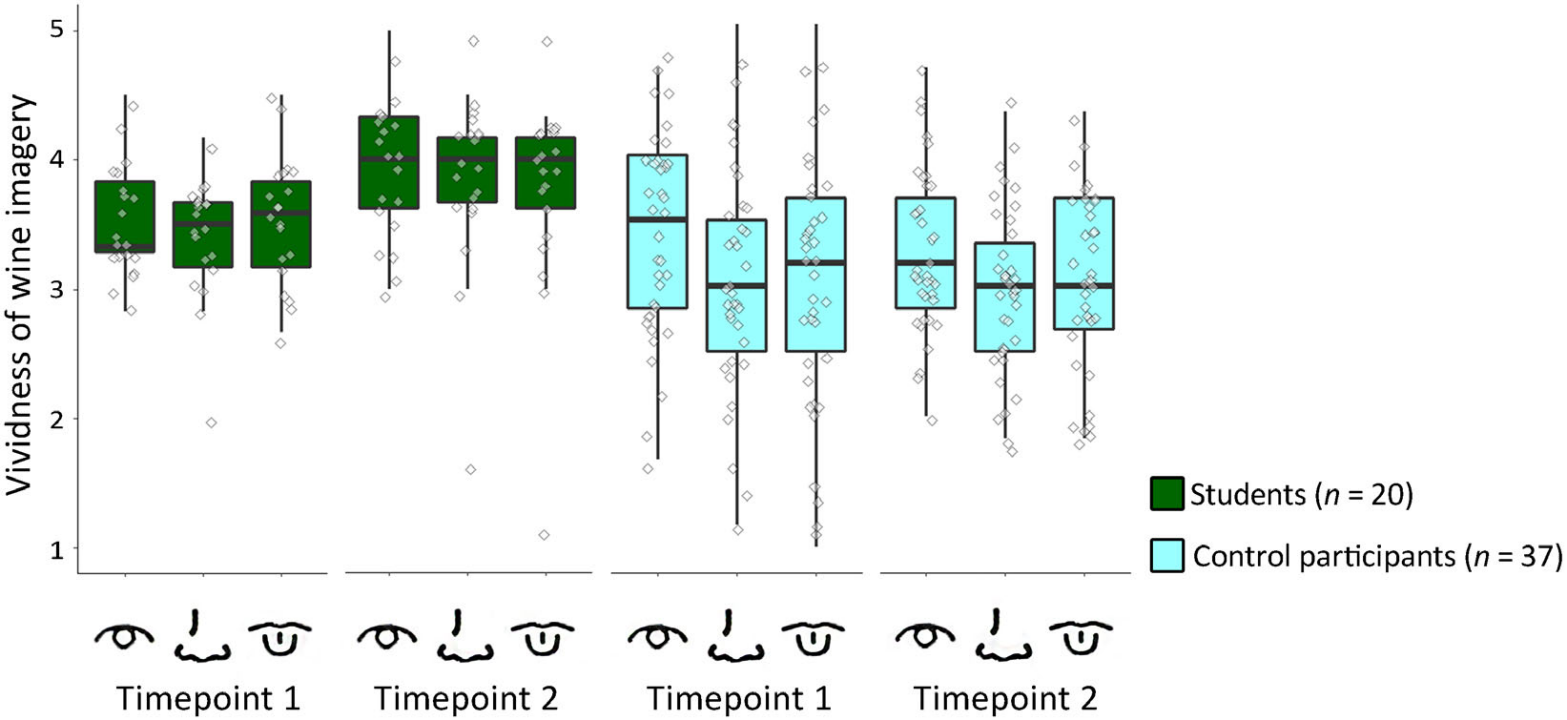
Boxplots for ratings on the VWIQ at T1 and T2 for students and control participants, for Study 2. Note: Box‐and‐whisker plots display median, first and third quartiles ranges, and whiskers indicate range of the data. Diamonds portray individual data points. Icons represent the imagery modalities: color, smell, and taste.

Nevertheless, during the 6‐month period that students received practical training in wine tasting as part of the professional wine course they attended, their vividness of imagery for wine increased. Using a mixed ANOVA with Group (students, controls) as a between‐participants factor and Modality (color, odor, taste), and Time (T1, T2) as within‐participant factors on VWIQ, we found students had more vivid imagery than controls, *F*(1, 55) = 12.08, *p* = .001, $\eta_{p}^{2}$ = 0.180. Critically there was a robust interaction between Group and Time, *F*(1, 55) = 8.35, *p* = .006, $\eta_{p}^{2}$ = 0.132. Pairwise comparisons showed that before training, the difference between students (*M* = 3.50, *SD* = 0.68) and controls (*M* = 3.16, *SD* = 0.68) was not significant, *p* = .080, Cohen's *d* = 0.50; but after training, students (*M = *3.86 *SD = *0.60) reported significantly more vivid wine imagery than controls (*M = *3.08, *SD = *0.60), *p* < .001, Cohen's *d* = 1.3 (see Fig. 3). Imagery also varied by Modality, *F*(2, 110) = 5.77, *p* = .004, $\eta_{p}^{2}$ = 0.095, but there was no interaction between Group and Modality, *F*(2, 110) = 2.05, *p* = .134, $\eta_{p}^{2}$ = 0.036, nor between Time and Modality, *F*(2, 110) = 0.385, *p* = .681, $\eta_{p}^{2}$ = 0.007, nor was there a significant three‐way interaction between Group, Time, and Modality, *F*(2, 110) = 2.22, *p* = .114, $\eta_{p}^{2}$ = 0.039. So training improved sensory imagery for wine across modalities.

We also compared students and control participants for their imagery of everyday odors and visual objects. We collected data for imagery of odors at two time points, just as with the VWIQ, so we analyzed the data for the VOIQ in the same manner. We found no effect of Group, *F*(1, 54) = 3.82, *p* = .056, $\eta_{p}^{2}$ = 0.065; Time, *F*(1, 54) = 2.92, *p* = .093, $\eta_{p}^{2}$ = 0.050; and no interaction between the two, *F*(1, 54) = 0.823, *p* = .368, $\eta_{p}^{2}$ = 0.015. So there was no general change in olfactory imagery for everyday odors as a result of wine training. Finally, we compared groups on their general visual imagery (VVIQ) at T2. This showed no general difference between students (*M* = 3.65, *SD* = 0.58) and controls (*M = *3.49, *SD* = 0.68), *t*(55) = 0.897, *p* = .373, *d* = 0.25, suggesting no difference in the ability to generate visual images of everyday scenes. Taken together, these results demonstrate that sensory imagery for an expert domain can become more vivid following training.

## 4 Discussion

While experts and novices reported similar vividness of imagery for everyday odors and visual scenes, they showed distinct response profiles for the imagery of wines. Specifically, wine experts had more vivid imagery for the color, odor, and taste of wines. Previous studies have found enhanced odor imagery with other types of expertise (Bensafi et al., 2013, 2017; Royet, Delon‐Martin, & Plailly, 2013), but this is the first study to show the same holds for wine experts. Critically, simple knowledge of wines, found in both experts and students before onset of training, did not lead to more vivid wine imagery. Instead, we found that practical training was necessary to enhance imagery in wine experts.

Wine experts work with wines on a daily basis and odors and tastes are particularly important. For example, wine experts often create wine‐food pairings, imagining how the tastes and textures of food match those of a wine (Harrington, 2005; Spence, 2020). In addition, when recognizing and interpreting wines, experts recall specific prototypes of wines (Hughson & Boakes, 2002), which may be seen as a form of imagery. These are also the types of activities students engage in to become wine experts, thereby shaping their ability to imagine aspects of wine (cf. Tempere et al., 2019).

Training affected the ability to imagine the color, odor, and taste of wine, but not everyday objects. This is in line with previous proposals for how musical training affects sound imagery, where students became better at imagining the pitch and acoustic character of sounds, but not visual objects (Aleman et al., 2000). This also dovetails with other findings on wine expertise, focusing on memory (Croijmans, Arshamian, Speed, & Majid, under review; Zucco, Carassai, Baroni, & Stevenson, 2011) and language (Croijmans & Majid, 2016), indicating the superior skills experts display do not extend beyond their domain of expertise (cf. Kimball & Holyoak, 2000). Taken together, this suggests mental imagery of the senses is malleable, and that perceptual modalities can become more important with experience (cf. Majid, Speed, Croijmans, & Arshamian, 2017).

It has been suggested that odor imagery is critically embodied (Arshamian, Manko, & Majid, 2020). For example, blocking sniffing can impair the quality of an imagined odor for those who are good odor imagers, but not for poor odor imagers (Arshamian, Olofsson, Jönsson, & Larsson, 2008; Bensafi, Pouliot, & Sobel, 2005); odor imagery training improves odor sensitivity (Tempere, Hamtat, Bougeant, de Revel, & Sicard, 2014); and odor imagery can interfere with the perception of real odors (Djordjevic, Zatorre, Petrides, & Jones‐Gotman, 2004). Imagery could be improved through mere exposure to perceptual stimuli, such as merely experiencing the smell and flavor of wine (cf. Arshamian & Larsson, 2014). One way this could happen is through perceptual learning where experts become more attentive to particular aspects of a stimulus, and thereby sharpen their mental representations of relevant perceptual features (Goldstone, 1998; Walk, 1966; White et al., 2020). This improvement could be restricted to stimuli to which people are frequently exposed, and therefore may not transfer to other stimuli even in the same perceptual modality (cf. Spence, 2019; Spence & Wang, 2019).

As an alternative possibility, Bensafi and colleagues hypothesize that practice may enhance verbal and emotional associations, as well as changing sensorimotor components, with the effect that richer semantic associations are more easily activated during imagery, for example, the imagined *shiraz* wine may simultaneously activate semantic concepts of “bold,” “Australia,” “red,” “vanilla,” and so on (Bensafi et al., 2013, 2017). Our study does not distinguish these accounts, but having established that training enhances wine imagery, future studies could attempt to tease these apart. For example, if fMRI showed activation of language and emotion networks, as well as perceptual networks during imagery in wine experts, then this would be evidence in favor of the latter account.

The present study treated mental imagery for the color, smell, and taste of wine as distinct modalities, although imagery—similar to perception itself—likely works in a cross‐modal fashion (Spence & Deroy, 2013), such that imagery in one modality leads to enhanced activation in another. This connected nature of the senses offers interesting opportunities for future studies, given the highly multisensory nature of wine pairing and other culinary arts (cf. Spence, Youssef, & Deroy, 2015).

We used self‐report measures to tap into mental imagery which risks the possibility that differences between groups or modalities are due to response biases rather than experienced imagery. However, it is difficult to explain how response bias would lead to the particular pattern of sensory imagery we find, for example, experts reporting enhanced imagery for wine odors but not everyday odors. Moreover, previous studies have shown that self‐reported vividness of mental imagery is related to fMRI BOLD activity in sensory‐specific areas of the brain (Huijbers, Pennartz, Rubin, & Daselaar, 2011; Olivetti Belardinelli et al., 2009), and as Pearson, Deeprose, Wallace‐Hadrill, Heyes, and Holmes (2013) suggest, odor imagery questionnaires are a useful and valid way to assess mental imagery abilities.

We found that students enrolled in a professional wine course had enhanced imagery for wine after 6 months, even though imagery is given no explicit role in the curricula of most wine courses. This stands in stark contrast to imagery in other types of expertise; for example, professional sports, where motor imagery is used to improve performance (Weinberg, 2008). Our study shows that without specific imagery training, wine imagery was enhanced; other studies have shown that direct training of imagery can also improve the skill (cf. Royet et al., 2013). For example, training has been shown to improve sensitivity for particular odors (Tempere et al., 2014, 2019). Given our results, the role of training in imagery could be explored further. For example, a study with naïve participants who engage in a training procedure similar to that employed by Tempere, Cuzange, Bougeant, Revel, and Sicard (2012) or alternatively with blind‐taste training (cf. Wang & Prešern, 2018) could explore the effect of interference of imagined smells on the recognition of learned odors (similar to Djordjevic, 2004; Djordjevic et al., 2004).

Our results also suggest imagery training could be incorporated into wine courses to help students hone their skills further. Imagery training could be deployed as a tool for individuals learning to distinguish and describe wines, and to combine wine and food, and so further improve the efficacy of wine education. Additionally, other flavor domains, such as for coffee (Van Doorn, Wuillemin, & Spence, 2014), beer (Van Doorn, Watson, Timora, & Spence, 2020), and tea (Wan et al., 2014), may also benefit from including imagery in their curricula. It is possible that these different flavor domains have different implications for imagery across modalities, since visual properties may play a different role in each (compare, for example, the appreciation of color in the domain of wine which uses glassware to coffee in opaque coffee cups; cf. Carvalho & Spence, 2018; Van Doorn et al., 2014).

To conclude, we show that experience has a pronounced effect on mental imagery, underscoring the considerable plasticity in multisensory mental imagery—and cognition in general—which demonstrates the importance of considering human behavior in its diverse contexts. Furthermore, a focus on imagery lends itself to interesting possibilities for future research and application within the domain of food and drink.

## Authors' contributions

IC, LS, AA, and AM conceived of and designed the studies. Data collection and data analysis were performed by IC with analysis checked by LS. Selection of tests and interpretation were performed by all the authors. IC drafted the manuscript with critical edits by AA, LS, and AM. All the authors approved the final version of the manuscript for submission.

## Open practices statement

Neither of the studies reported in this article was formally pre‐registered. The SPSS data, including SPSS syntax, as well as questionnaires used in these experiments, have been made available via Github: https://github.com/ICroijmans/WineImagery.

### Open Research badges

This article has earned Open Data and Open Materials badges. Data and materials are available at https://github.com/ICroijmans/WineImagery.

## Notes

^1^ Flavor—the multisensory experience of what is perceived in the mouth—is closely related to smell (Small & Prescott, 2005; Spence, Smith, & Auvray, 2015) and uses overlapping physiological mechanisms (Rozin, 1982; Shepherd, 2006). When people talk about taste, they usually mean the multisensory experience of flavor (Rozin, 1982; Small & Prescott, 2005). In Dutch—the language in which participants were tested—there is no linguistic distinction between *taste* and *flavor* (“smaak”). Here, we use “taste” to encompass this distinction.

^2^ See https://www.wsetglobal.com/qualifications/wset‐level‐4‐diploma‐in‐wines

^3^ See https://www.wijnacademie.nl/vinologenopleiding
